# Cranial base and maxillary changes in patients treated with Frankel’s functional regulator (1b)

**DOI:** 10.4317/medoral.17631

**Published:** 2012-02-09

**Authors:** Juan J. Alió-Sanz, Carmen Iglesias-Conde, Jose Lorenzo-Pernía, Alejandro Iglesias-Linares, Asunción Mendoza-Mendoza, Enrique Solano-Reina

**Affiliations:** 1 DDS, MS, PhD. Professor of orthodontics, Complutense University of Madrid, Spain; 2 DDS, PhD.Private practice, Orense, Spain; 3 DDS , PhD.Associate professor, Department of orthodontics, Complutense University of Madrid; 4 DDS , MSc, PhD.Lecturer Masters Programme in Orthodontics and Dentofacial Orthopaedics School of Dentistry. University of Seville; 5 DDS , MSc, PhD.Professor of paedriatic dentistry, University of Seville. Spain; 6 DDS , MSc, PhD.Chairman of orthodontics, University of Seville. Spain

## Abstract

Objectives: The purpose of this study was to assess cranial base and maxillary growth in patients with Class II-type I malocclusions when treated with Frankel’s functional regulator (FR-1b). Study Design: The treatment group was made up of 43 patients that were divided into two groups: prepubescent (n: 28), and pubescent (n: 15). The control group included 40 patients who did not receive any kind of treatment and were likewise divided into a prepubescent group (n: 19), and a pubescent group (n: 21). A computerized cephalometric study was carried out and superimpositions were done in order to assess the antero-posterior, vertical and rotational movement of the maxilla. Results: The results indicate that anterior cranial length is not affected by the regulator but the cranial deflection of the treatment group was diminished. Although a slight counterclockwise rotation effect on the upper jaw was observed due to treatment, no growth restriction of the maxilla in a vertical or antero-posterior direction was observed compared to other non-treated Class II-type I malocclusion patients. Conclusion: The functional regulator does not have any effect on anterior cranial length, but it does affect the angulation of the cranial base. According to our results, the appliance has demonstrated a flattening effect of the cranial base (p<0.05) in the treated sample. The functional regulator induces counterclockwise rotation rather than vertical or sagittal changes in the maxilla.

** Key words:**Orthodontics, frankel regulator, class II treatment, cephalometry, superimposition.

## Introduction

Class II-type 1 malocclusion is a common clinical problem in orthodontics, with approximately 15%-30% of North American children and 20%-30% of all orthodontic patients having this type of dentoskeletal imbalance (1) and represents approximately 50% of all orthodontic treatment in a European representative sample (2). Of the various treatment strategies for Class II treatment, muscular and presumable skeletal regulation through Frankel’s 1b appliance has been for decades one of the most widely used treatments for Class II malocclusions in prepuberal children. Despite this protocol being so widespread, there is relatively little information in the literature about the dentoskeletal changes of this 2-phase nonextraction Class II therapy in adolescents or young adults in relation to other skeletal structures besides those changes that take place in the mandibular bone (3,4).

The aim of this cephalometric study was to evaluate the role of upper jaw skeletal modifications on the outcomes of this type of nonextraction Class II therapy as well as other potential effects on the cranial base between groups.

Whether the functional regulator (FR) induces a stimulatory effect on lower jaw growth in Class II type I patients (5-8) or whether it just forces a reaccommodating anterior positional change (9) has not yet been fully clarified. Most short-term and long-term studies done to date have found an increase in anterior mandibular growth in patients treated with a FR compared to patients in control groups (6,7). Along these lines, some authors (10) observed a statistically significant increase in mandibular length due to FR treatment. Nevertheless, an elegant study carried out by McNamara et al. (11) has questioned whether Class II corrective results were just the expression of an anterior positional change rather than an effect of increased mandibular length.

On the other hand, the upper jaw is another skeletal structure potentially modified in FR Class II treatments. Studies on normal craniofacial growth showed a downwards and forwards maxillary growth pattern with substantial interindividual variation (12,13). Findings on FR effects on the maxilla are often contradictory. Many studies defended the idea that anterior maxillary growth is restricted during treatment, (14-17) while others have noticed a downwards redirection of upper jaw growth inducing a clockwise slope in the palatal plane (7,16,18,19). Still other authors have found no effects on the upper jaw due to FR Class II treatment (11,20-22).

Whether the FR induces a stimulatory effect on cranial base growth on Class II-type I patients or whether it causes changes in the cranial base slope in growing patients has not yet been determined.

## Material and Methods

In order to assess any potential change in skeletal structures of the maxilla and the cranial base due to FR treatment, a retrospective cephalometric study was performed in Class II-type I malocclusion patients.

Sample

A total of 83 Caucasian individuals were selected consecutively for four years for inclusion in the study because they came to the Department of Orthodontics of the Complutense University of Madrid for dental screening. Of the total number of patients, only 43 patients (18 boys/25 girls) met the following inclusion-exclusion criteria for being included in the treated sample (FR-group): 1) Class II-type I malocclusion; 2) No craniofacial abnormalities; 3) Subspinal to nasion to supramenton (ANB) angle or convexity equal to or greater than 5º; 4) Non-dolichofacial growth pattern; 5) No hypodontia or dental inclusions or extractions; 6) No previous maxillofacial surgery; 7) Treatment with a functional regulator type-Ib exclusively; 8) Treatment period between the ages of 8-14 years old; 9) Caucasian origins. The control sample (Ct-group) included 40 patients (22 boys/18 girls) with identical inclusion-exclusion criteria but who have not undergone any kind of orthodontic treatment. These patients refused orthodontic treatment but were admitted to take part in the growth study performed by the Department of Orthodontics of the Complutense University of Madrid. FR and Ct-groups were both divided in two additional subgroups, prepubescent (8-11 years old; n:28; 12 boys-16 girls) and pubescent (12-14 years old; n:15; 6 boys - 9 girls) in order to compare different growth stages. FR group: prepubescent (n:28; 12 boys-16 girls) and pubescent (n:15; 6 boys - 9 girls). Ct-group: prepubescent (n:19; 10 boys-9 girls) and pubescent (n:21; 12 boys - 9 girls).

Functional regulator appliance

The FR was constructed according Frankel’s design (5,23,24). The construction bite was obtained using a direct functional chew-in technique in neutrocclusion with 2-4mm wax height. The average treatment period was 1year and 6months. Instructions were given to use the appliance for 1hour/day for the first 15days, 3hours/day for the next 15days, then in addition to the three hours during the day to wear the appliance at night for one month, and finally to use the appliance all day and night.

Cephalometric records 

**Figure 1:**
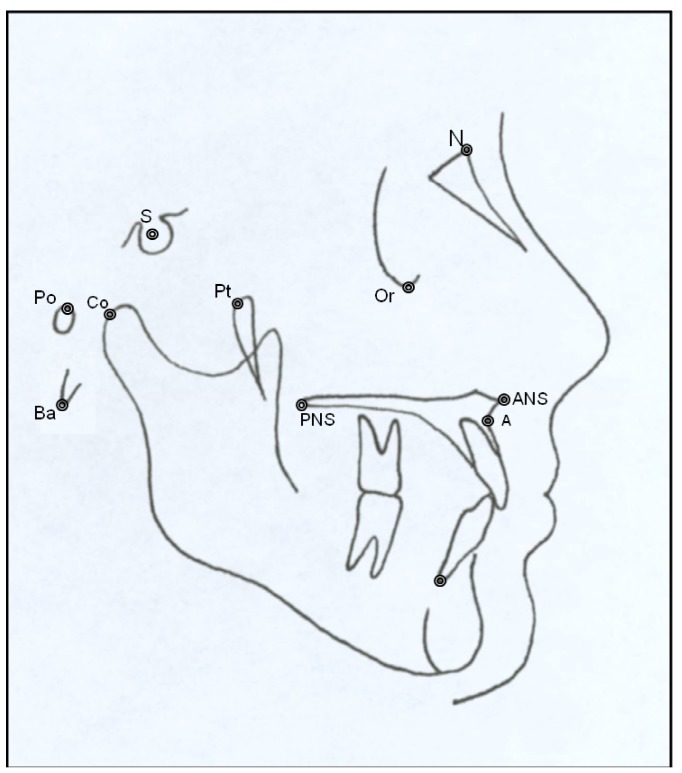
Cephalometric measurements used in this study. N(nasion), S(sella turcica), Ba(basion), Cf(pterygomaxillary), Po(porion), Or(suborbital), A(point A), ANS(anterior nasal spine), PNS(posterior nasal spine), Co(condylion).

**Figure 2:**
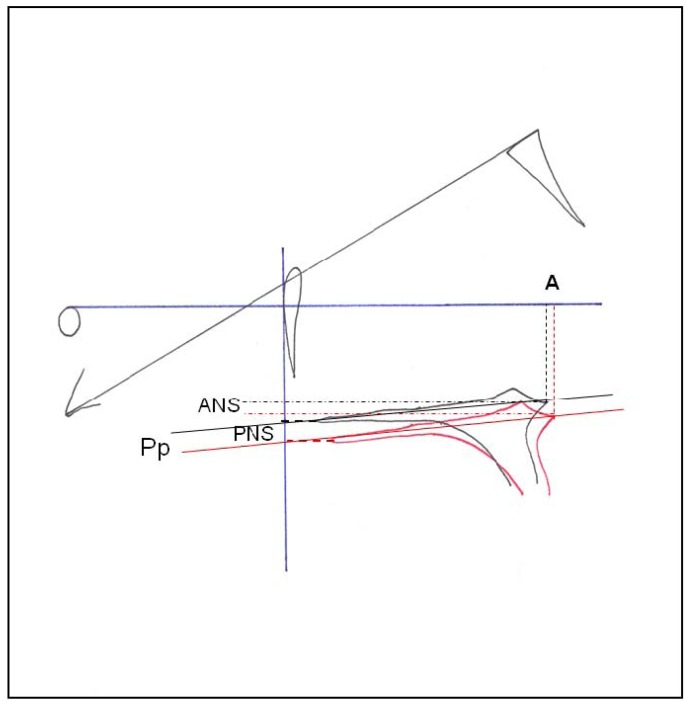
Superimposition of the maxilla. Ba-N plane at Nasion point. Anterior cranial length(CC-N): distance between CC and nasion.; Cranial deflection(N-Ba/Po-Or): angle formed between the basion-nasion plane and the Frankfurt plane; SNA angle: angle formed by the sella turcica-nasion(S-N) and nasion-point A(N-A) planes; Maxillary depth(Po-Or/N-A): angle formed by the Frankfurt plane and the N-A plane; Distance from point A to the nasion perpendicular to Frankfurt (A-FHp): distance between point A and a line perpendicular to the Frankfurt plane (Po-Or) that descend from the nasion(N); Effective maxillary length(Co-A): distance from the highest and most posterior part of the condylion(Co) to the maximum concavity of the anterior maxillary outline(point A); Maxillary height: angle formed by the nasion-Cf and Cf-point A planes, where Cf is defined as the intersection of the pterygoid plane(PtV) and the Frankfurt plane; Slope of the palatal plane(Po-Or/ANS-PNS): angle formed by the Frankfurt plane and palatal plane; A: the maximum concavity of the anterior maxillary outline(point A); ANS: anterior nasal spine; PNS: posterior nasal spine; Pp: palatal plane.

Lateral x-rays were obtained with a Siemens-Palomex-OY x-ray machine, and cephalometric tracings were done with the NemotecDental-Studio (v.2.0.0.1) orthodontic software with reference to the landmarks shown in (Fig. 1). The lines and angles described in (Fig. 2) were traced for the comparative measurements. All cephalometric measurements were performed by two independent researchers following same criteria (25).

Reliability of the method

All the cephalometries were traced by two experimented researchers (J.A.S. and C.I.C.) belonging to the general research project on growth carried out in the Master’s Program in Orthodontics at the Universidad Complutense of Madrid. These researchers calibrate their measurements annually to avoid any error in the cephalometric tracings. In order to estimate the intra-examiner variation for the radiological evaluation all the radiographs were evaluated twice by the same experienced examiner (J.A.S.). In order to estimate the inter-examiner variation all the radiographs were evaluated by a second experienced examiner (C.I.C.).

Once both researchers have performed the tracings, they were compared to each other thereby obtaining one of three distinct possibilities:

1) Type I Concordance: total coincidence of the tracings.

2) Type II Concordance: difference in some parameter among the tracings that are less than the following values: Anterior cranial length(CC-N): Less than 1mm; Cranial deflection(N-Ba/Po-Or): Less than 1º; SNA angle: Less than 30; Maxillary depth(Po-Or/N-A): Less than 30; Distance from point A to the nasion perpendicular to Frankfurt (A-FHp): Less than 1mm; Effective maxillary length(Co-A): Less than 3mm; Maxillary height: Less than 1mm; Slope of the palatal plane(Po-Or/ANS-PNS): Less than 10.

3) Type III Concordance: Greater difference than described above.

In Type II concordance the arithmetic mean is established between the two parameter values that do not coincide. When the difference is greater (Type III concordance) the tracings are done again and are referenced against the three concordance possibilities mentioned above. The causal error was determined using Dahlberg’s formula (S.E.=√‾d²/2n) and the systematic error using a t test for a P<0.05.

Statistical Analysis

Two-way analysis of variance (ANOVA) with interaction and the Student’s t-test for independent samples (p<0.05) were then obtained to determine whether there was any interaction between age and the treatment, if age affects the treatment and if the treatment has any effect or not on the variable. The Student t-test was used to compare the FR-group and Ct-group in the prepubescent and pubescent subgroups after verifiying randomness, using the Student´s t test for independent samples (the Wald-Wollowitz runs test at p>0.05 for all variables in both groups) and for normality (the Shapiro-Wilk test for normality at p>0.05 for all variables in both groups).

## Results

Cranial Base

No significant differences were found between the Ct-group and the FR-group in anterior cranial length ( Table 1). Similarly, no significant differences were obtained between groups according to age either. However, significant differences (p<0.05) were found in the prepubescent group in relation to cranial deflection (Ba-N/FH) between the FR and Ct-groups. Surprisingly, the treatment group showed a flatter cranial base than the control group, while no such tendency was observed for the pubescent group. Despite no significant differences being found in the angular variable, both genders displayed obvious significant differences in anterior cranial length measurements ( Table 1).

**Table 1:**
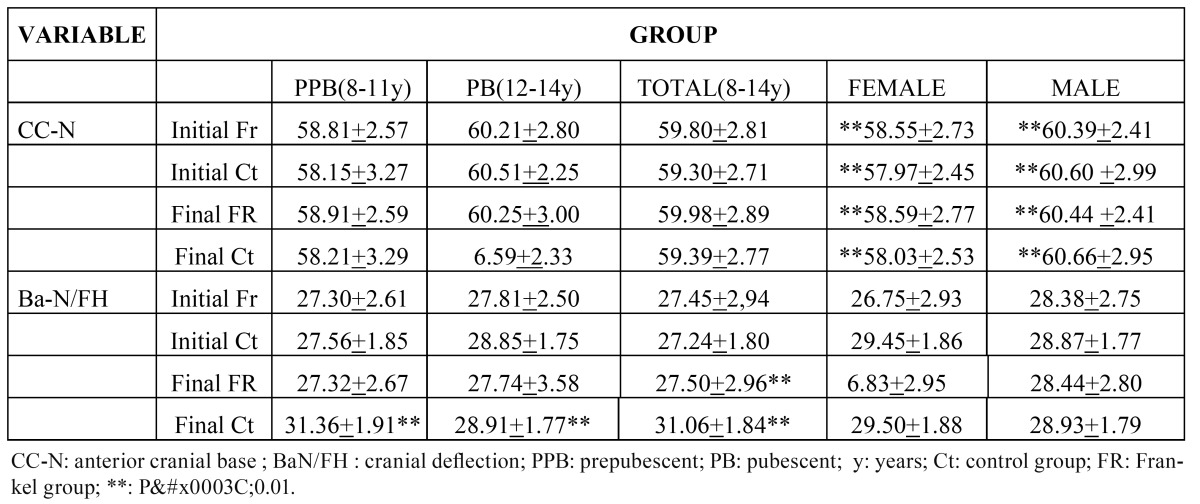
Cephalometric measurements of the cranial base according to age and sex.

Maxilla

While the slope of the palatal plane showed significant differences in the prepubescent group of the FR-group compared to that of the Ct-group, none of the other selected measurements of the upper jaw showed significant differences between groups ( Table 2). Interestingly, the treatment group ended up having a more parallel palatal plane in relation to the Frankfurt plane compared to the Ct-group, which showed a notable clockwise rotation of the palatal plane. Remarkably, no such differences were noted for the pubescent group.

**Table 2:**
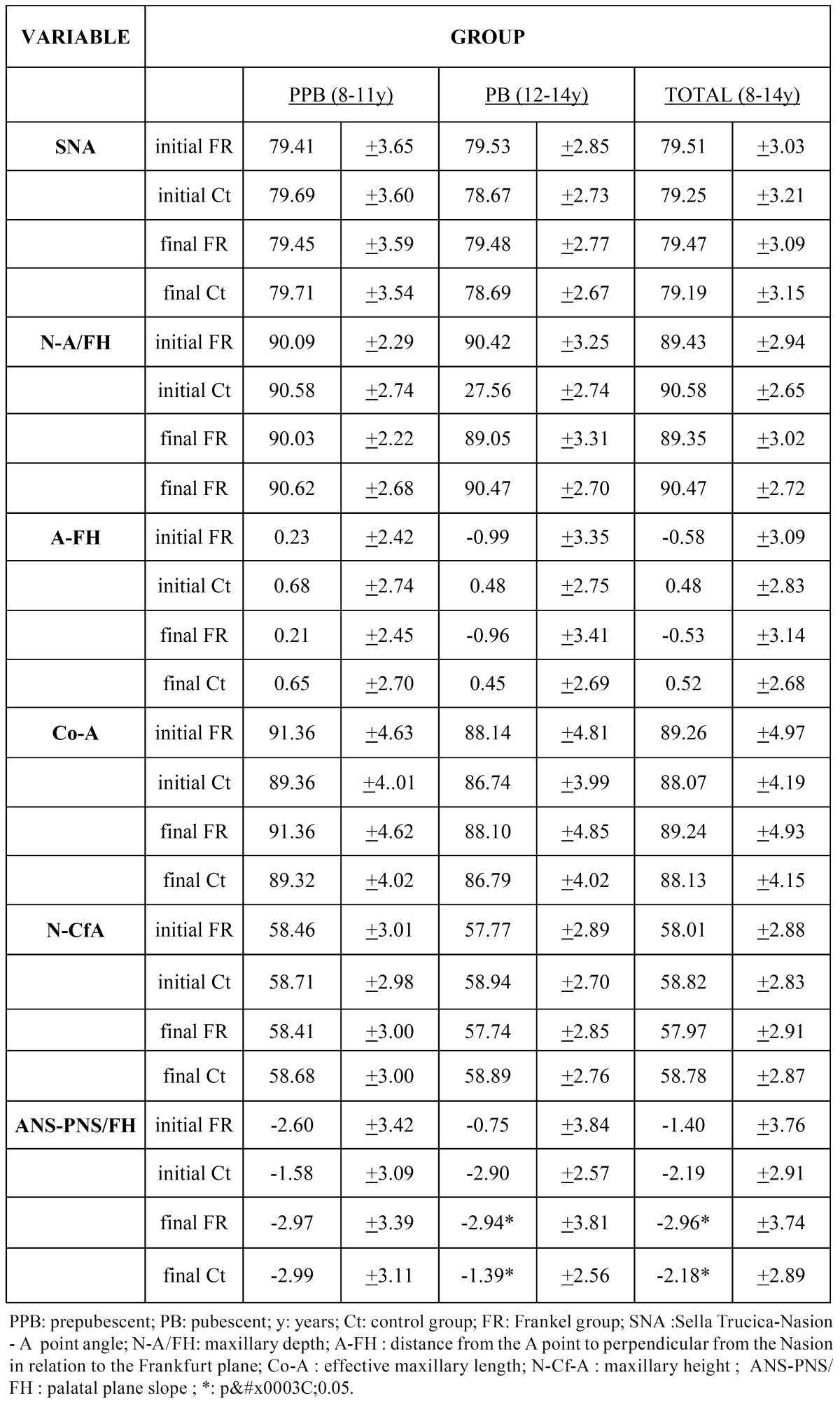
Cephalometric measurements of the maxilla.

According to sex, none of the variables showed differences within each group. Nevertheless, expected significant differences were only found between the boys and girls, in the Ct-group as well as in the FR-group, in relation to the effective maxillary length measurements (Co-A) ( Table 3).

**Table 3:**
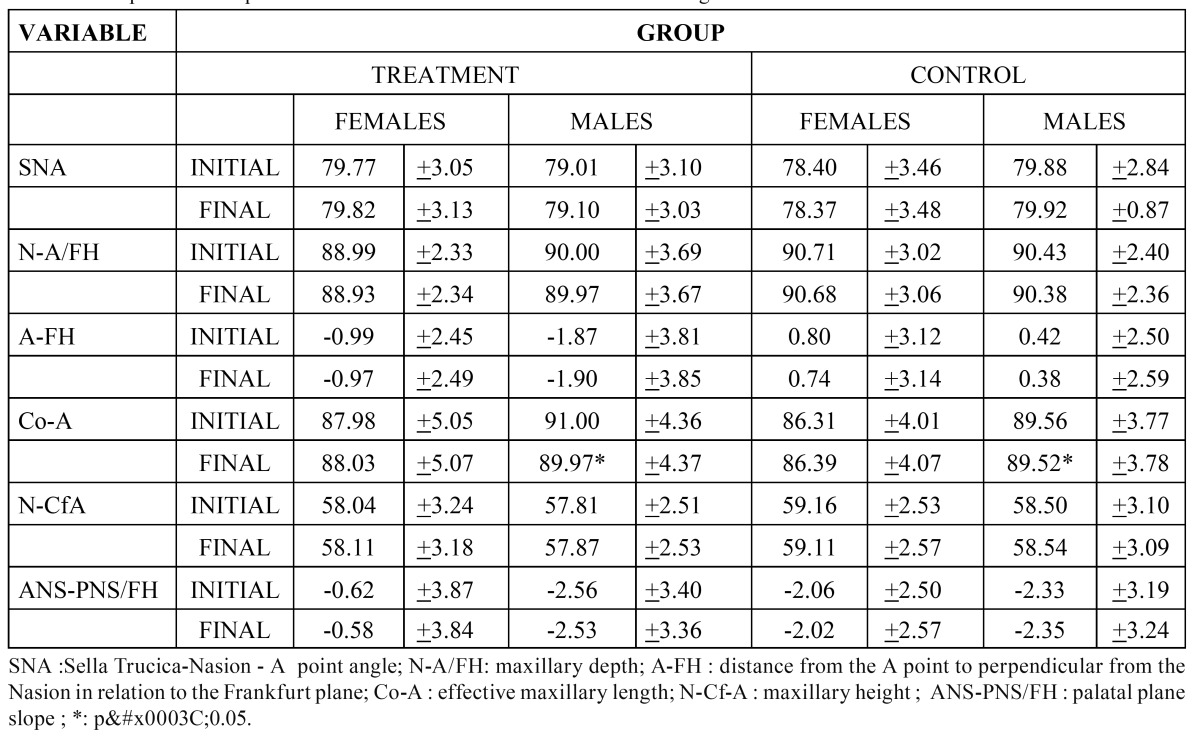
Comparison of cephalometric measurements of the maxilla according to sex.

Changes were seen in the sagittal, vertical and rotational planes in the maxillary superimpositions. Sagittally, measurements related to the point A position showed significant differences between the FR-group and the Ct-group ( Table 4). The point A position was farther back in the prepubescent stage of the FR-group. However, these differences returned to normal in the pubescent stage. Regarding the vertical measurements, our study of the ANS and PNS did not show any significant differences between the two groups ( Table 4). Rotational plane measurements displayed significant differences between the FR-group and the Ct-group, both in the overall averages as well as in the prepubescent and pubescent groups ( Table 4). Notably, these differences reflected a counterclockwise rotation of the palatal plane in the FR-group while no sex variability was found for any of the variables analyzed ( Table 4).

**Table 4:**
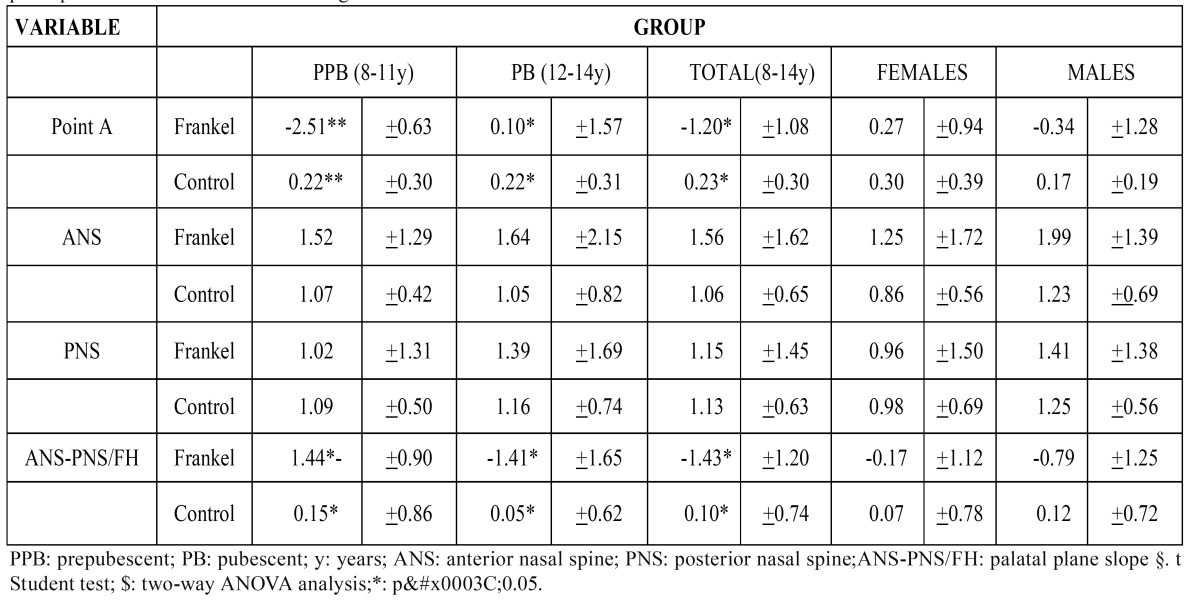
Superimpositions of the Maxilla-Sagital plane, Maxilla-Vertical plane< Maxilla-Rotational plane. Comparison of superimpositions of the maxilla according to sex.

## Discussion

Effects of the FR on the cranial base

The anterior cranial base length did not show any differences among the groups studied, not even in the prepubescent and pubescent groups, which leads us to state that the anterior cranial base is definitely not affected by treatment with a FR. According to Björk (26), the increase of cranial vault size is very small after the age of 10-12 years, while the facial and mandibular bones continue growing until after the age of 20 years.

As shown in our results, a flatter cranial base is observed in the prepubescent FR-group. In that way, changes in the Ba-N or Po-Or planes would result in a flattening of the cranial base. Despite a natural change in Nasion remodeling and growth also being capable of inducing this result, it might be reasonable to suggest that this result may be due to the effect of the appliance. Some authors (27) have described a 0.86mm displacement of the Basion towards the back with a cranial base rotation of 0.44º (NSBa). These researchers suggested that the effect observed in the cranial base is due to the posterior movement of the Basion rather than an anterior movement of the Nasion. Although significant differences on displacement of up to 2.5mm were found among some patients, according to some authors, this could be the result of a great variability in the position of the Basion rather than the effect of the appliance on the cranial base (27).

Effects of the FR on the maxilla

Sagittal Changes

The FR appliance resulted in little or null effect on the upper jaw structure. Even though significant differences are found related to the point A position in the different projections between the FR-group compared to the Ct-group, no sagittal growth restriction can be attributed to the appliance. The functional appliance does not restrict antero-posterior maxillary growth.

The absence of restrictive effects on the maxilla is extremely important since it readily points us to using the functional appliance when the maxilla is retruded with an open nasolabial angle (23).

Notable controversy exists regarding the effect of the FR appliance on the maxilla. Thus, while many studies, (10,21,27-30) including ours, indicated that there is no appreciable antero-posterior effect on this structure, others have found restrictive properties of the FR on upper jaw growth (14,15).

Restricted upper jaw growth, or the “headgear effect”, found by other authors might result from appliance design variations, such as a lack of interproximal reduction of the molars as indicated by McNamara et al. (11), or due to a one-stage construction bite, averaging more than 5.9mm of mandibular advancement, as noted by Falck et al. (19). These authors suggest that such substantial mandibular advancement might produce a stretching of mandibular retrusive muscles causing upper jaw restriction. In addition, Owen (14) has suggested a shortening effect in mandible muscles during patients’ sleep. The protrusive muscles, such as the lateral pterygoid, allow the retrusive muscles, like the posterior temporal, to retrude the mandible to its normal position at rest. According to this author’s hypothesis, this muscular pressure is transmitted to the upper jaw through the appliance and brings about an effect similar to headgear. The muscular force generated produces a functional force vector which is the cause of the “headgear” effect on the maxilla (5). Nevertheless, this author suggested that the slight maxillary retrusion observed could at least partially occur due to the notable degree of individual variability among the study subjects.

According to our results of the point A superimpositions, a -1.20mm posterior displacement of the maxilla is observed in the FR-group, compared to that of the Ct-group. As expected, the Ct-group showed anterior maxillary growth of 0.23mm throughout the period of the study. Remarkably, such restrictive effects on upper jaw growth are just observed in the prepubescent group.

The significant difference of 1.43mm anterior displacement found between the FR and Ct-groups in the superimposition of point A might be explained by previous studies (11,23). These authors believe this appliance has little or no effect on upper jaw growth. According to these authors’ hypothesis, observed differences might be caused by point A landmark variability. In that way, point A would be easily modified by the radicular position of the upper central incisors through a remodeling mechanism. Therefore, a change in the lingual slope of the upper incisors can have a small but significant effect on cephalometric maxillary measurements.

Another explanatory factor that may contribute to such observed differences is that suggested by Nielsen (16). According to this author, the cephalometric point A would be affected by the clockwise rotation of the upper jaw which in turn moves backwards at the end of treatment.

Vertical changes 

The FR appliance used in this study did not produce any effect on the vertical displacement of the maxilla during treatment. The upper jaw grows vertically at the same rate and in the same direction in both groups, as shown by the anterior nasal spine (ANS) and posterior nasal spine (PNS) superimpositions and even by the maxillary height measurement (N-Cf-A). None of these measurements showed significant differences compared to the Ct-group. Similarly, previous studies seem to support our results (11,16,29).

Rotational changes 

In order to determine the presence or absence of maxillary rotation we used Ricketts’ measurement of the slope of the palatal plane (Po-Or/ANS-PNS) and the distance between the initial and final positions of the palatal plane. The prepubescent group showed significant differences in so far as a more parallel palatal plane to the Frankfort plane is observed in the FR-group compared to the Ct-group. In contrast, the Ct-group experienced a slight clockwise rotation of the palatal plane. According to these results, the FR-group showed a slight counterclockwise rotation in the initial and final palatal plane superimpositions.

Though the FR may tend to parallelize the palatal plane in a counterclockwise direction, when we compare the initial and final superimpositions of this group we find that there is a clear counterclockwise rotation in this plane, while in the control group there is practically no rotation of the palatal plane when we consider the data referring to total measurements. If we look at the data regarding the prepubescent group, we see that in this group there is a clockwise rotation of the palatal plane. Contrary to the results obtained in this study, other authors have found that the palatal plane in the group treated with the functional appliance showed a clockwise rotation (8,30).

## Conclusions

As can be inferred from the study results we can conclude that.

1. According to our results, the FR appliance has demonstrated a flattening effect of the cranial base(p<0.05) in the treated sample but it does not have any effect on anterior cranial length.

2. The FR does not produce any growth restriction of the maxilla in an antero-posterior direction.

3. The FR appliance does not modify normal vertical maxillary growth compared to other non-treated Class II-type I malocclusion patients.

4. A slight counterclockwise rotation effect on the upper jaw is observed due to FR treatment.
